# Factors influencing cerebrospinal fluid and plasma HIV-1 RNA detection rate in patients with and without opportunistic neurological disease during the HAART era

**DOI:** 10.1186/1471-2334-7-147

**Published:** 2007-12-21

**Authors:** Paulo P Christo, Dirceu B Greco, Agdemir W Aleixo, Jose A Livramento

**Affiliations:** 1 Eduardo de Menezes Hospital, FHEMIG, Belo Horizonte, MG, Brasil; 2 Department of Infectious Diseases, Federal University of Minas Gerais, Brasil; 3 Department of Neurology, University of São Paulo, Brasil

## Abstract

**Background:**

In the central nervous system, HIV replication can occur relatively independent of systemic infection, and intrathecal replication of HIV-1 has been observed in patients with HIV-related and opportunistic neurological diseases. The clinical usefulness of HIV-1 RNA detection in the cerebrospinal fluid (CSF) of patients with opportunistic neurological diseases, or the effect of opportunistic diseases on CSF HIV levels in patients under HAART has not been well defined. We quantified CSF and plasma viral load in HIV-infected patients with and without different active opportunistic neurological diseases, determined the characteristics that led to a higher detection rate of HIV RNA in CSF, and compared these two compartments.

**Methods:**

A prospective study was conducted on 90 HIV-infected patients submitted to lumbar puncture as part of a work-up for suspected neurological disease. Seventy-one patients had active neurological diseases while the remaining 19 did not.

**Results:**

HIV-1 RNA was quantified in 90 CSF and 70 plasma samples. The HIV-1 RNA detection rate in CSF was higher in patients with neurological diseases, in those with a CD4 count lower than 200 cells/mm^3^, and in those not receiving antiretroviral therapy, as well as in patients with detectable plasma HIV-1 RNA. Median viral load was lower in CSF than in plasma in the total population, in patients without neurological diseases, and in patients with toxoplasmic encephalitis, while no significant difference between the two compartments was observed for patients with cryptococcal meningitis and HIV-associated dementia. CSF viral load was lower in patients with cryptococcal meningitis and neurotoxoplasmosis under HAART than in those not receiving HAART.

**Conclusion:**

Detection of HIV-1 RNA in CSF was more frequent in patients with neurological disease, a CD4 count lower than 200 cells/mm^3 ^and detectable plasma HIV-1. Median HIV-1 RNA levels were generally lower in CSF than in plasma but some patients showed higher CSF levels, and no difference between these two compartments was observed in patients with cryptococcal meningitis and HIV-associated dementia, suggesting the presence of intrathecal viral replication in these patients. HAART played a role in the control of CSF HIV levels even in patients with cryptococcal meningitis and neurotoxoplasmosis in whom viral replication is potentially higher.

## Background

The assessment of plasma viral load has led to revolutionary advances in the understanding of viral dynamics in the HIV-infected organism and has become one of the most important tools for monitoring the response to antiretroviral (ARV) therapy [1-4]. Highly active antiretroviral therapy (HAART) has brought a real chance of effectively controlling the infection, with high suppression of plasma viral load and improvement of the cellular immune response, thus reducing mortality and the incidence of new opportunistic infections and HIV-associated dementia (HIV-D) [5-11]. However, opportunistic neurological diseases continue to occur, especially in developing countries. In Brazil, despite a public health system that provides ARV drugs free of charge, this disease continues to occur due to irregular immunovirological monitoring and late diagnosis of the infection [12].

Evidence indicates that in different compartments of the body such as the central nervous system, HIV replication can occur relatively independent of systemic infection [13-18], and intrathecal replication of HIV-1 has been observed in patients with HIV-related and opportunistic neurological diseases. In contrast to plasma viral load, the usefulness and predictive value of the analysis of cerebrospinal fluid (CSF) viral load are less clear [19,20].

The clinical usefulness of HIV-1 RNA detection in the CSF of patients with opportunistic neurological diseases, or the effect of opportunistic diseases on CSF HIV levels in patients under HAART has not been well defined. Many studies regarding CSF viral load have been conducted in developed countries on patients with HIV-related neurological disease; however, few studies have been carried out in developing countries and on patients with opportunistic disease.

The objectives of the present study were to compare HIV RNA levels in CSF and plasma of HIV-infected patients with toxoplasmic encephalitis, cryptococcal meningitis, HIV-D and without active neurological diseases, as well as to determine the characteristics that led to a higher detection rate of HIV RNA in CSF and the influence of HAART on CSF viral load.

## Methods

We prospectively evaluated 90 HIV-infected patients who were submitted to lumbar puncture between May 2002 and May 2003 as part of the work-up for a suspicion of neurological disease at a public AIDS Reference Hospital (Eduardo Menezes Hospital, FHEMIG), Belo Horizonte, Brazil. The study was approved by the Research Ethics Committee of the institution and written informed consent was obtained from all participants.

Data collected from each patient included gender, age, CD4+ lymphocyte count, use of ARV therapy, duration of ARV therapy, and presence and type of neurological disease.

Neurological diseases were diagnosed based on the following criteria: criteria of the Working Group of the American Academy of Neurology Task Force [21] for the diagnosis of HIV-D and vacuolar myelopathy, a suggestive skull computed tomography scan and a clinical and tomographic image response to specific drug treatment for the diagnosis of toxoplasmosis, and a positive India ink result, a specific cryptococcal antigen test or positive CSF culture for the diagnosis of cryptococcal meningitis. Tuberculous meningitis was diagnosed based on clinical-neurological signs of lymphocytic meningitis and the presence of alcohol-acid resistant bacilli or a positive CSF culture. Stereotactic biopsies were obtained for the diagnosis of progressive multifocal leukoencephalopathy (PML) and bacterial abscess. An undetermined diagnosis was considered when the patients showed neurological syndromes characterized by meningitis, encephalitis or expansive intracranial lesions and when no etiology could be established after work-up.

CSF samples from all 90 patients were submitted to the quantification of HIV-1 RNA and the virus was also quantified in plasma in 70 of them. Twenty patients didn't have a plasma sample collected or samples were collected 48 hours after the CSF samples, so they were excluded from the analysis.

CSF and plasma samples were collected within an interval of 48 hours between each other and stored at -70°C until the time of processing within a maximum period of 6 months. HIV-1 RNA was quantified by NASBA using 1.0 ml centrifuged plasma and 0.1 ml not centrifuged CSF according to manufacturer instructions (Nuclisens HIV-1 QT, Organon TeKniKa, Boxtel, Netherlands), with a sensitivity of 80 copies/ml (1.90 log_10 _copies/ml). None of the CSF samples contained more than 10 red cells/mm^3^.

### Statistical analysis

Individual associations of the variables CD4 T lymphocyte count, use of ARV therapy, presence of neurological disease and detectable plasma HIV-1 RNA with the detection of HIV-1 RNA in CSF were determined by the chi-square test. Multivariate logistic regression analysis was used to evaluate the overall effect of these four variables. The nonparametric Wilcoxon test was used for comparison of CSF and plasma viral load. The nonparametric Mann-Whitney test was applied to the comparison of CSF viral load in patients with and without ARV therapy. The level of significance was set at 5% for all tests.

A log10 transformation was performed on all HIV-1 RNA concentration values (copies per milliliter). For patients with undetectable HIV-1 RNA, a log-scale value of 0 was assigned to avoid the problem of expressing zero logarithmically. All statistical analyses were performed with the SPSS 8.0 program (SPSS Inc.).

## Results

### Characteristics of the population

Table 1 summarizes the clinical and laboratory characteristics of the 90 patients. The median CD4+ T lymphocyte count was 89 cells/mm^3 ^and no differences in median CD4 count were observed between patients with different types of neurological diseases or between those undergoing ARV therapy or not. Forty-nine (54.4%) of the 90 patients used ARV drugs, all in the HAART regimen.

**Table 1:** Characteristics of the 90 subjects studied

Mean age [years (range)]	36 (20–60)
Sex (n)	
Male	65
Female	25
CD4-positive cells (cells/mm^3^)	
Mean	149
Median	89
Range	1–405
Mean time since diagnosis [month (range)]	24 (0.5–108)
Pre-existing ARV therapy (n)	
Yes	49
No	40
Mean duration of ARV therapy [month (range)]	10 (0.3–60)
Neurologically asymptomatic (n)	19
Neurologically symptomatic (n)	71
HIV-associated dementia	6
Vacuolar myelopathy	1
Opportunistic CNS infections (43)	
Toxoplasmosis	19
Cryptococcal meningitis	15
Tuberculosis meningitis	3
PML	2
Associations	
Cryptococcal and tuberculous	3
Cryptococcal and toxoplasmosis	1
Cryptococcal and vacuolar myelopathy	1
Other (stroke, brain abscess, neuropathy)	4
Undetermined neurological diagnosis	16

ARV, antiretroviral drug; CNS, central nervous system;

PML, progressive multifocal leukoencephalopathy

Seventy-one (78.9%) of the 90 patients presented with neurological diseases, while the remaining 19 patients (21.1%) did not have active opportunistic or AIDS-related neurological diseases and no clinical or laboratory evidence of active infection was observed in these patients.

### Detection rate of HIV RNA in CSF

HIV-1 RNA was detected in 62.2% of the 70 plasma samples and in 55.6% of the 90 CSF samples analyzed. The detection rate of RNA HIV-1 in CSF was higher in patients with neurological diseases (63.4%), in patients with a CD4+ T cell count lower than 200 cells/mm^3 ^(64.4%), in patients not undergoing ARV therapy (82.5%), and in patients with detectable plasma HIV-1 RNA (71.4%). However, after multivariate analysis the use of ARV was no longer significant in the detection of HIV-1 RNA in CSF (Table 2).

**Table 2:** Frequency of HIV-1 RNA detection in CSF according to the clinical and laboratory characteristics of the patients

Characteristic	Presence (n)	CSF HIV-1 RNA		p (univariate)^b^	p (multivariate)^c^	OR
					
		>80 (%)^a^	<80 (%)			
Disease^d^	Yes (71)	63.4	36.6	< 0.001	0.0010	22.1
	No (19)	26.3	73.7			
CD4	<200 (17)	64.4	35.6	< 0.001	0.0021	18.7
	>200 (73)	17.6	82.4			
ARV use	No (40)	82.5	17.5	< 0.001	NS	*
	Yes (49)	34.7	65.3			
Plasma viral load	>80 (56)^a^	71.4	28.6	< 0.001	0.0008	22.6
	<80 (14)	14.3	85.7			

^a^Detection limit: 80 copies/ml; ^b^chi-square test; ^c^multivariate logistic regression analysis; ^d^neurological disease; ARV, antiretroviral drug; NS, not significant; OR, odds ratio.

### Viral load in CSF and plasma of the 70 subjects

**Figure 1:**
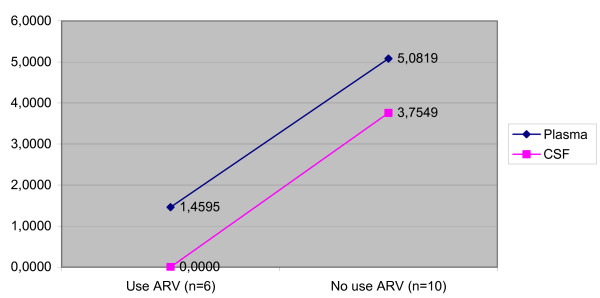
Median viral load CSF and plasma in the patients with toxoplasmic encephalitis according to antiretroviral therapy.

**Figure 2:**
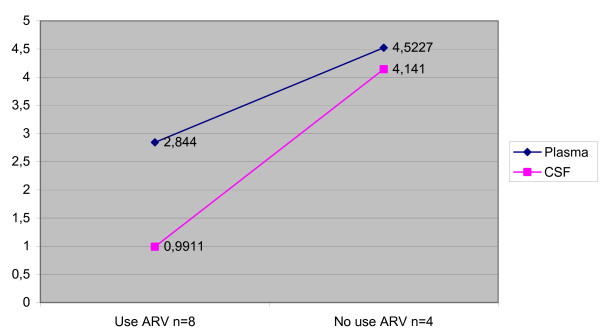
Median viral load CSF and plasma in the patients with cryptococcal meningitis according to antiretroviral therapy.

**Figure 3:**
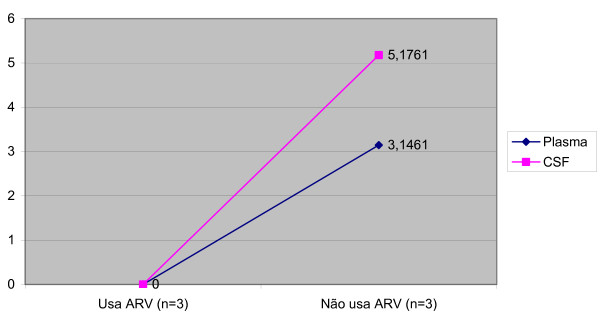
Median viral load CSF and plasma in the patients with Dementia-HIV according to antiretroviral therapy.

**Figure 4:**
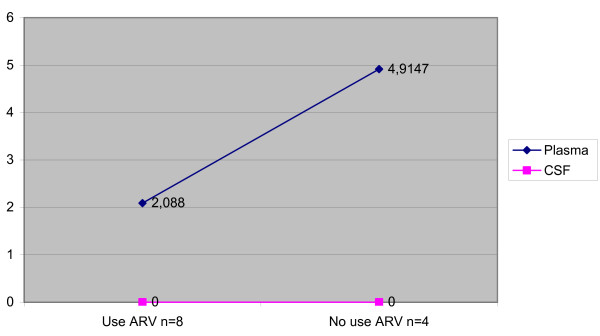
Median viral load CSF and plasma in the patients with no neurological disease. according to antiretroviral therapy.

Median viral load was lower in CSF than in plasma in the total population (p < 0.001), in patients without neurological diseases (p = 0.012), and in patients with toxoplasmic encephalitis (p = 0.013) (Table 3). In the patients with toxoplasmic encephalitis, there was a difference between CSF and plasma viral load, but just in the patients that didn't use therapy. There weren't any differences in the patients that used therapy (Figure 1). On the other hand, no significant difference between the two compartments was observed in patients with cryptococcal meningitis (p = 0.209) and HIV-D (p = 0.273) and this occurred in patients that did or didn't use therapy (Figures 2 and 3). Concerning the patients that didn't have neurological disease, there was a difference of viral load between compartments in just the patients that used therapy (p = 0,043) (Figure 4).

**Table 3:** Median viral load in CSF and plasma of 70 patients with and without neurological disease

**Patient group**	**Copies/ml (median)**		
	
	**Plasma**	**CSF**	**p**^**a**^
**Total population (n = 70)**			
VL (absolute values)	19,200	695	
Log_10_	4.28	2.81	< 0.001
**Neurological disease (n = 58)**			
VL (absolute values)	22,200	1,850	
Log_10_	4.35	3.27	< 0.001
**Without neurological disease (n = 12)**			
VL (absolute values)	335	1	
Log_10_	2.45	<1.9	0.012
**Neurotoxoplasmosis (16)**			
VL (absolute values)	27,500	3,200	
Log_10_	4.44	3.45	0.013
**Cryptococcal meningitis (n = 12)**			
VL (absolute values)	16.500	4.900	
Log_10_	4.22	3.70	0.209
**Undetermined diseases (n = 13)**			
VL (absolute values)	120,000	17,000	
Log_10_	5.08	4.23	0.028
**HIV-D (n = 6)**			
VL (absolute values)	2,641	910	
Log_10_	2.82	2.89	0.273

^a ^nonparametric Wilcoxon test; VL, viral load; HIV-D; HIV-associated dementia.

In absolute values, the median plasma and CSF viral load ratio was approximately 28:1, and this ratio was lower in patients with neurological diseases (12 times) and higher in patients without neurological diseases (335 times). The lowest ratio was observed in patients with cryptococcal meningitis (3.4 times) and HIV-D (3 times). Twelve (17%) of the 70 patients for whom concomitant CSF and plasma samples were available showed higher absolute values in CSF than in plasma, with 11 of them having opportunistic disease of the central nervous system and HIV-D. The mean and median number of cells in CSF was higher in these patients than in the total population.

Median CSF and plasma viral load were significantly lower in patients with opportunistic diseases in general, cryptococcal meningitis and neurotoxoplasmosis under HAART. Among the 35 patients with opportunistic neurological diseases in whom HIV-1 RNA was detected in CSF, 20 received HAART (median of <1.9 log_10 _copies/ml) and 15 did not (median of 3.76 log_10_). In plasma, the median was 2.82 log_10 _copies/ml for patients receiving HAART and 4.57 log_10 _copies/ml for those who did not.

## Discussion

### Detection rate of HIV-1 RNA in CSF

In the present study, the detection rate of the virus in CSF (56%) and plasma (62%) was lower than those reported in other investigations. One possible explanation is that 54% of our patients were on HAART and 21% of the patients did not have HIV-related or secondary neurological diseases, with neurological diseases being known to increase viral replication in CSF and plasma and the consequent detection of the virus [22-25].

Qualitative studies using PCR have shown that HIV can be detected in CSF at frequencies ranging from 64% [26] to 90% [27]. A variable frequency has also been reported in other studies using quantitative detection methods depending on patient characteristics, with frequencies ranging from 66% [23,28,29] to 98% of CSF samples. The latter rate was reported by Gisslen *et al.*[30] for patients not undergoing ARV therapy who used a more sensitive PCR kit with a detection limit of 20 copies per ml.

Studies regarding HIV-1 RNA detection in CSF should be analyzed with caution. First, quantitative methods have been shown to be less sensitive than qualitative methods [31]. Second, some studies showing a higher detection rate of HIV-1 RNA in CSF were performed before 1998, when most patients were not on HAART. Third, the groups of patients analyzed in these studies differ in terms of clinical characteristics such as time since infection, presence of HIV-related or secondary neurological disease, or absence of symptoms. Finally, in the case of quantitative methods, the sensitivity of the method used might influence the detection rate of HIV-1 in CSF.

Detection of HIV-1 RNA in CSF varied according to the characteristics of the population and was higher in patients with a CD4 count lower than 200 cells/mm^3^, patients with neurological disease, patient not using ARV drugs, and patients with detectable plasma HIV-1 RNA, but use of ARV therapy was no longer significant upon multivariate analysis.

The chance of detecting HIV-1 RNA in CSF of patients with neurological diseases was approximately 22 times higher than in patients without neurological diseases. In patients with a CD4 count lower than 200 cells/mm^3 ^the chance was almost 19 times higher when compared to patient with a CD4 count higher than 200 cells/mm^3^, and a 23 times higher chance of detecting HIV-1 RNA in CSF was observed in patients with detectable plasma viral load.

The unfavorable effect of neurological diseases on CSF HIV levels has also been shown to be independent of ARV therapy and may depend both on the increased permeability of the blood-brain barrier, associated with enhanced trafficking of infected cells and cytokines during neurological disease, and on the increased local production of HIV-1 despite systemic antiviral control [15]. However, median CSF viral load was lower in patients with cryptococcal meningitis and neurotoxoplasmosis receiving HAART than in those with cryptococcal meningitis and neurotoxoplasmosis who did not use HAART, demonstrating that HAART plays a certain role in the control of CSF viral load in these patients with potential intrathecal viral replication.

HIV-1 RNA levels in CSF have been shown to be correlated with opportunistic neurological diseases [24,25] and HIV-D [22], and HIV-1 RNA is more easily detected in patients not undergoing HAART [15,32]. HAART has been shown to be effective in reducing viral replication in both plasma and CSF [15,33-35].

### Viral load in CSF and plasma

Significant differences in median viral load between the two compartments (plasma and CSF) were also observed in the total population studied and in patients with and without neurological diseases, but there was no difference when individuals with cryptococcal meningitis and HIV-D were considered separately and this occurred in patients that did or didn't use therapy. The ratio between median plasma and CSF viral load was lower in these patients, showing that HIV-1 RNA levels increased in the two compartments, but the increase was more significant in CSF, probably due to intrathecal replication of HIV-1 or to increased passage of the virus through the blood-brain barrier in these patients. The difference between CSF and plasma viral load is considered to be an independent sign of viral replication in the cerebral compartment [36,37].

In absolute values, the median viral load was 28 times higher in plasma than in CSF, but in 12 (17%) of the 70 patients for whom concomitant CSF and plasma viral loads were available, HIV levels were higher in CSF than in plasma. These findings are similar to reports in the literature showing that median or mean plasma levels are usually higher than the corresponding concentrations in CSF, but CSF viral load can be higher in individual patients [25,29,33,38-42]. CSF HIV-1 RNA levels are often relatively low, approximately 1 log lower than plasma levels [43]. The finding that 17% of the patients had a higher viral load in CSF than in plasma supports the theory that HIV levels in CSF do not merely reflect plasma HIV levels.

## Conclusion

Detection of HIV-1 RNA in CSF was directly related to the characteristics of the patients such as the presence of neurological disease, a CD4 count lower than 200 cells/mm^3^, and detectable plasma HIV-1 RNA. Median HIV-1 RNA levels were generally lower in CSF than in plasma but some patients showed higher CSF levels, and no difference between these two compartments was observed in patients with cryptococcal meningitis and HIV-D, suggesting the presence of intrathecal viral replication in these patients. HAART played a role in the control of CSF HIV levels even in patients with cryptococcal meningitis and neurotoxoplasmosis in whom viral replication is potentially higher.

## Competing interests

The author(s) declare that they have no competing interests.

## Authors' contributions

PC participated with subject evaluations and planning and participated in analysis, interpretation of the data and in the study planning. JL helped in the study planning and draft the manuscript, participated in analysis. AA participated in analysis and coordinated the viral load assays. DG helped in the study planning and draft the manuscript, participated in analysis.

## Pre-publication history

The pre-publication history for this paper can be accessed here:


